# The* Mycobacterium tuberculosis* CRISPR-Associated Cas1 Involves Persistence and Tolerance to Anti-Tubercular Drugs

**DOI:** 10.1155/2019/7861695

**Published:** 2019-04-02

**Authors:** Jiawei Wei, Nan Lu, Zhiying Li, Xuanyan Wu, Tao Jiang, Li Xu, Chun Yang, Shuliang Guo

**Affiliations:** ^1^Chongqing Medical University, Chongqing 400016, China; ^2^The First Affiliated Hospital of Chongqing Medical University, Chongqing 400016, China

## Abstract

Tuberculosis remains one of the leading causes of death worldwide. Even if new antitubercular drugs are currently being developed, the rapid emergence and spread of drug-resistant strain remain a severe challenge. The CRISPR associated proteins 1 (Cas1), a most conserved endonuclease which is responsible for spacer integration into CRISPR arrays, was found deleted in many specific drug-resistant strains. The function of Cas1 is still unknown in* Mycobacterium* type III-A CRISPR family. In this study, the Cas1 (Rv2817c) defect was found in 57.14% of clinical isolates. To investigate the function of Cas1 in new spacer acquisition, we challenged* Bacillus Calmette–Guérin *(BCG) with a mycobacteriophage D29. Newly acquired spacer sequence matches D29 genome was not found by spacer deep-sequencing. We further expressed Cas1 in recombinant* Mycobacterium smegmatis*. We found that Cas1 increased the sensitivity to multiple anti-tuberculosis drugs by reducing the persistence during drug treatment. We also showed that Cas1 impaired the repair of DNA damage and changed the stress response of* Mycobacterium smegmatis*. This study provides a further understanding of Cas1 in* Mycobacterium tuberculosis complex* (MTBC) drug-resistance evolution and a new sight for the tuberculosis treatment.

## 1. Introduction

Tuberculosis is caused by* Mycobacterium tuberculosis *(MTB). It remains one of the top ten causes of death worldwide and the leading cause from a single infectious agent [1]. The emergence and spread of multidrug-resistant* M. tuberculosis* (MDR-MTB) and extensively drug-resistant* M. tuberculosis* (XDR-MTB) strains have worsened this scenario [2]. Moreover, the development of new and effective anti-tuberculosis drugs continues to be challenging [3]. Therapy using mycobacteriophage which specifically infects mycobacteria has become a novel treatment of MDR-MTB and XDR-MTB. But the resistance to mycobacteriophage can rapidly arise during phage therapy, which is still the main obstacle to phage therapy [4]. The resistance of* Mycobacterium tuberculosis complex* (MTBC) to phage involves a variety of mechanisms including surface changes [5], lysogenic immunity, toxin-antitoxin systems [6], and clustered regularly interspaced short palindromic repeats (CRISPR)–CRISPR associated proteins (Cas) systems [7].

The CRISPR–Cas system provides an adaptive immunity against phage via spacer-encoded CRISPR RNAs that are complementary to invasive nucleic acids, and it has been found in 90% of archaea and 40% of bacteria [8]. Most species contain two or more CRISPR loci which comprise short direct repeats, spacers, and leader. Foreign DNA derived from phage genome or invasive plasmid is stored in unique spacer sequences and inserted into CRISPR arrays, which endow the CRISPR-Cas system with the acquired immunity of the past encounters [9]. Recently a strong bias in the phage genome locations where the spacers were derived has been observed in many CRISPR subtypes that confer the immunity to phage [10–12].* Mycobacterium tuberculosis,* a Gram-positive genus bacteria belonging to Actinobacteria, contains a type III-A CRISPR family [9, 13]. Up to now, the spacer adaptation has not been observed in type III-A systems [14], and none of the spacer elements in* M. tuberculosis* CRISPR array can perfectly match the known mycobacteriophage sequences [15]. The function of type III-a system in* M. tuberculosis* is worthy of further study.

CRISPR associated proteins 1 (Cas1), the most conserved protein in Cas family, has been proved to be a metal-dependent DNA-specific endonuclease in many genera of bacteria and responsible for spacer integration into CRISPR arrays [16–19]. Cas1 from different genera of bacteria displays related but different biochemical properties, and it is also labeled multifunctional because it involves DNA repair in* Escherichia* [17]. However, it is noted that the Cas1 is a dispensable adaptation module in type III-A [18]. Beijing family of MTBC has deleted Cas1, yet it is still a very successful pathogen in high tuberculosis load areas and represents about half of the clinical isolates in Far-East-Asia [20]. Moreover, MTBC Beijing family strain has been observed to have an increased tendency to be MDR [21]. Although the deletion of Cas1 is found to be associated with drug resistance in many genera of bacteria [22–25], it is still unclear if Cas1 in MTBC relates to drug-resistance evolution. The transcription of Cas1 gene in MTBC varies in response to antibiotics treatment and other environmental stresses, which suggests Cas1 may play a role in drug resistance and stress response [26].

In this study, we focus on the function of type III-A CRISPR system and Cas1. First we explored the function of type III-A CRISPR in the acquisition of phage genome by using mycobacteriophage D29 and* Bacillus Calmette–Guérin *(BCG) which has a similar type III-A CRISPR system with* M. tuberculosis* [27]. New spacer acquisition was not observed according to the CRISPR spacer deep-sequencing. Then we further expressed Cas1 in* M. smegmatis* which is congenital absence of Cas1 by using mycobacterial shuttle vector pMV261. The expression of Cas1 in* M. smegmatis* impaired DNA damage repairing and also increased susceptibility to multiple antibiotics and environmental stresses.

## 2. Materials and Methods

### 2.1. Bacteria Strain, Plasmid, and Growth Conditions

*Escherichia coli*, BCG, and* M. smegmatis* mc^2^ 155 were provided by the China General Microbiological Culture Collection Center. Mycobacterial shuttle plasmid pMV261 was obtained from Beijing Tuberculosis and Thoracic Tumor Research Institute. The genome of* M. tuberculosis* H37Rv was provided by the respiratory laboratory of the First Affiliated Hospital of Chongqing Medical University. Middlebrook (MB) 7H9 broth supplemented with 0.5% (v/v) glycerol and 0.05% (v/v) Tween80 was used to culture BCG,* M. smegmatis* mc^2^155 and* M. tuberculosis*. Luria-Bertani medium was used to culture* E. coli* strains. For maintenance of the pMV261 plasmid, kanamycin was added to a final concentration of 50*μ*g/ml.

### 2.2. Mycobacteriophage Challenging and Spacer Deep-Sequencing

Monoclonal of BCG was grown in 7H9 medium with 0.5%(v/v) glycerol, 10% OADC(v/v), and 0.05%(v/v) Tween 80, followed by incubation at 37°C. When OD_600_ reached 0.7 mycobacteriophage D29 was used to infect BCG with initial phage–host ratios of 10:1 and 2:1, respectively. 2ml was taken from each culture every 48h, and then OD_600_ values and plaque forming units (PFU) were measured. Two CRISPR loci DNA from bacterial cultures were amplified by PCR, and then PCR content (20*μ*l) was electrophoresed for 30 min on a 2% (wt/vol) agarose gel. Following gel separation, the expanded band was excised from the gel and purified using the DNA clean-up kit (Tiangen, Beijing). Each PCR product was loaded on an Illumina HiSeq instrument according to manufacturer's instructions (Illumina, San Diego, CA, USA) at Genewiz Inc., Suzhou. New spacer insertions were called based on sequence alignments of the resulting reads.

### 2.3. Detection of Cas1 Gene (Rv2817c) in Clinical Isolates

The clinical isolates of* M. tuberculosis* were obtained from newly diagnosed pulmonary tuberculosis patients from June 2017 to December 2017. Mycobacterial colonies on positive Lowenstein-Jensen culture medium were suspended in 500 ml of distilled water and heated for 20 min at 95°C and then centrifuged for 5 min at 12,000 ×g. Next, the bacterial DNA containing supernatant was amplified using forward primer MtbCas1F and reverse primer MtbCas2R. The PCR products were analyzed using 2% (wt/vol) agarose gel.

### 2.4. The Construction of Recombinant* M. smegmatis*

All the primers used in the study are shown in Suppl.  Table 1. The gene of Cas1 was amplified from* M. tuberculosis* H37Rv by PCR using the forward primer EcoCas1F containing a His-tag and reverse primer HindCas1R. Then the PCR product of approximately 1066bp was cloned into mycobacterial shuttle plasmid pMV261. The constructed plasmid and empty pMV261 vector were confirmed by DNA sequencing (Tsingke, China) and transformed into* M. smegmatis* mc^2^ 155 by electroporation [28]. The transformants were screened by PCR using the above primer pairs. The confirmed strain harboring with pMV261-Rv2817c was named as mc^2^ 155_Cas1 and the strain harboring with empty pMV261 vector was denoted as mc^2^ 155_Vec. The expression of His-tagged Cas1 protein was detected by Western blotting using mouse anti-His antibody (TIANGEN, China).

### 2.5. Bacterial Growth Kinetics

Recombinant strains mc^2^ 155_Cas1 and mc^2^ 155_Vec were cultured in 7H9 medium until OD_600_ reached mid log-phase. Then two strains were reinoculated in fresh 7H9 medium at an initial OD_600_ of 0.02 and then cultured in 37°C orbital incubator (120 rpm) for 72 hours. 350*μ*l of cultures was taken at an interval of 4 hours for OD_600_ values determination.

### 2.6. Drug Susceptibility Assay

Drug sensitivity was determined using REsazurine Microtiter Assay (REMA) [29]. Antibiotics including isoniazid powder (Solarbio), rifampin powder (Solarbio), ethambutol powder (Solarbio), streptomycin solution 1mg/ml (Sigma), levofloxacin solution 1mg/ml (Solarbio), and ampicillin powder (Solarbio) were dispensed using serial dilutions in each well of a microplate suitable for fluorescence reading.* M. smegmatis* cultures were grown up to mid-exponential phase and then 100*μ*l exponentially growing* M. smegmatis* cultures were inoculated into 96-well plates containing 100*μ*l 7H9 medium complemented with drugs of different concentration in each well. The plates were sealed and incubated for 3 days at 37°C. Then 20 *μ*l of Alamar-Blue (Solarbio) was added to each well of the plates. After another day of incubation at 37°C, plates were read on a microplate reader (Varioskan LUX 3020-486, Thermo Fisher) to determine the relative fluorescence intensity (excitation 535nm and emission 590nm). Positive control (cells without antibiotic) was set to determine the maximum fluorescence that could be obtained, and well without cells was set as a negative control.

### 2.7. Viable Counts and Survival Curves

Exponentially growing* M. smegmatis* were diluted in 7H9 medium and then treated with agents, respectively. Subsequently,* M. smegmatis* were serially diluted and plated onto drug-free MB 7H10 agar plate. CFU counts were performed in triplicate by using a standard liquid droplet method. The percentage of survival was calculated relative to an untreated control sampled at the time when the agent was added.

### 2.8. Assessment of Persistence

10% exponentially growing* M. smegmatis* diluted in 7H9 medium was treated with antibiotics for 24h or 48h. Antimicrobial concentrations were as follows: rifampin 5*μ*g/ml; streptomycin 20*μ*g/ml; ethambutol 20*μ*g/ml; levofloxacin 20*μ*g/ml. CFU counts were performed in triplicate as previously described. Meanwhile, 20*μ*l of bacterial suspension was extracted and reinoculated in 180*μ*l fresh 7H9 medium supplemented with 20nM recombinant resuscitation-promoting factor E (RpfE) in 96-well microtiter plates to calculate most probable number (MPN). Plates were incubated at 37°C statically for 14 days until MPN calculated as standard procedure [30]. The persistence of* M. smegmatis* was assessed using the resuscitation index (RI), calculated as log 10 (MPN) -log 10 (CFU) [31].

### 2.9. Statistical Analysis

Data was analyzed by using Prism 7.0a (GraphPad Software). The results were expressed as mean ± SEM and analyzed by using Student's t-test for normally distributed data with equal variances.

## 3. Result

### 3.1. CRISPR Spacer Deep-Sequencing

Both MTB and BCG have the complete CRISPR loci and share the identical Cas1 (Rv2817c) gene which is conserved in most CRISPR loci of MTBC. Cas1 is an indispensable module for the adaptation process and the endonuclease activity of Cas1 plays a major role in new spacer acquisition [19]. Cas1 in* M. tuberculosis* Type III-A CRISPR belongs to the endonuclease super-family Cas1_I-II-III and shares the homologous domain architecture with the* E. coli* Type I-E and* Staphylococcus* Type III-A Cas1. Although the acquisition of foreign DNA has been observed in other members of super-family Cas1_I-II-III, the defensive function of* Mycobacterium* CRISPR-Cas system is still unclear.

**Figure 1:**
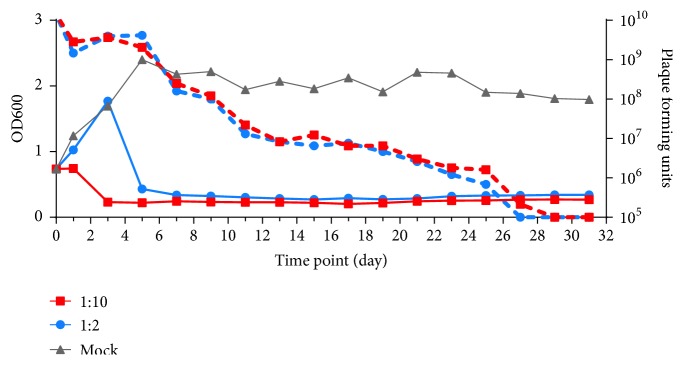
Dynamics of host–phage coexistence. The fluctuations of OD value (straight lines) and plaque forming units (dashed lines) in different MOI of 2 (blue) or 10 (red).

Overall, 12,861,726 sequencing reads from two experimental series across 31 daily samples were analyzed in this study. However, no newly acquired spacer sequence that matches mycobacteriophage D29 genome was found. Dynamics of host–phage coexistence was recorded (Figure 1). The population of bacilli dramatic decline after 1- or 3-day incubation, but BCG could not be completely eliminated by phage: host ratios of 2:1 nor 10:1 even after 60-day coculture. This result indicates the resistance of MTBC to D29 may not be due to the acquisition of foreign DNA from D29 genome. The Cas1 in* M. tuberculosis* Type III-A CRISPR may play a different role from the Cas1 in other genera.

### 3.2. Cas1 Gene (Rv2817c) Deletion Was Found in 57.14% of Clinical Isolates

Cas1 deletion is only observed in* M. tuberculosis* among all the known natural CRISPR-containing bacteria. The distribution Cas1 deletion strain also shows a strong regional bias associated with tuberculosis prevalence and antibiotic use [20]. In this study, a total of 35 clinical isolates of* M. tuberculosis* were obtained from newly diagnosed tuberculosis patients' sputum specimens from June 2017 to December 2017. Cas1 gene deletion was confirmed in 20 (57.14%) strains of clinical isolates by PCR (Suppl.  Figure 1), which is consistent with the previous studies of epidemiological prevalence of Tuberculosis. Cas1 deletion strain is frequently found in Far-East-Asia where there is a relatively high tuberculosis load and antibiotic pressure [20, 21]. This phenomenon indicates that the deletion of Cas1 may contribute to the spread of tuberculosis in these areas.

### 3.3. Expression of Cas1 Changes the Morphology and Growth Kinetic of* M. smegmatis*

**Figure 2:**
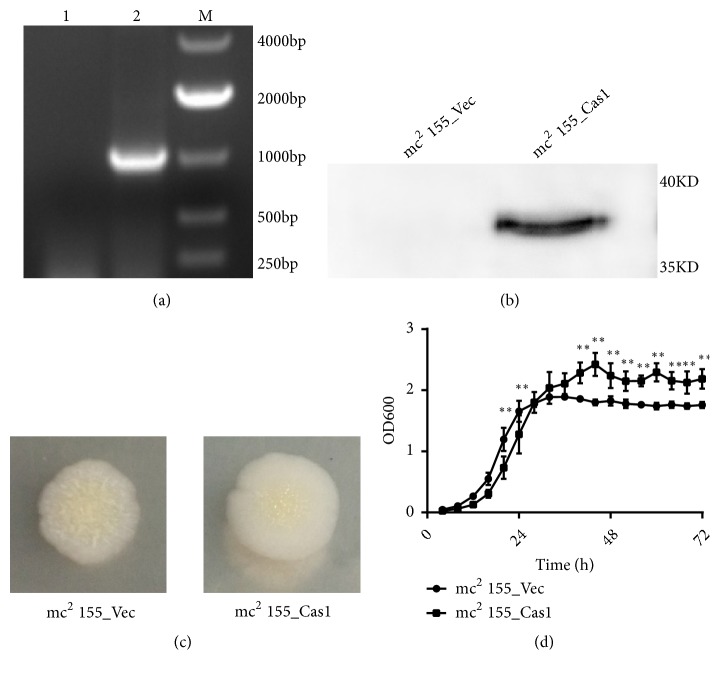
Expression of Cas1 changes the morphology and growth kinetic of* M. smegmatis.* (a) PCR amplification of Cas1 encoding gene (1016 bp) using mc^2^ 155_Vec (lane 1) and mc^2^ 155_Cas1 (lane 2). (b) Expression of Cas1 protein was measured by Western blotting of total lysate. (c) Morphological differences between mc^2^ 155_Vec and mc^2^ 155_Cas1 single clone grown on 7H10 agar plates. (d) Growth Kinetics of mc^2^ 155_Vec and mc^2^ 155_Cas1. Error bars indicate standard deviation of triplicate experiments. *∗* P<0.05; *∗∗* P<0.01.

The 38 kDa recombinant protein was detected in mc^2^ 155_Cas1 strain by Western blotting using mouse anti-His antibody, while it was absent in the total protein of mc^2^ 155_Vec strain (Figure 2(b)). When Cas1 was expressed, the colony of* M. smegmatis* became smoother and moister than mc^2^ 155_Vec strain (Figure 2(c)). The growth kinetics of two recombinant strains were measured as described above. The mc^2^ 155_Cas1 strain had a slower growth rate in exponential phase but a higher OD_600_ value in stationary phase (Figure 2(d)).

### 3.4. Cas1 (Rv2817c) Expression Increases* M. smegmatis* Sensitivity to Various Environmental Stresses

**Figure 3:**
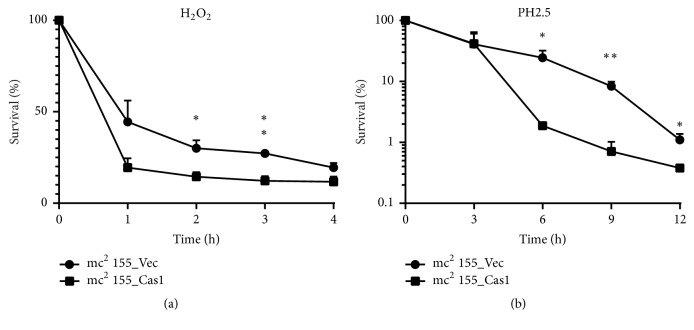
Cas1 recombinant* M. smegmatis* are hypersensitive to environmental stresses. Recombinant strains mc^2^ 155_Cas1 and mc^2^ 155_Vec were treated with (a) 5 mM H_2_O_2_ and (b) acidic MB 7H9 (pH 2.5) for indicated times. Error bars indicate standard deviation of triplicate experiments. *∗* P<0.05; *∗∗* P<0.01.

MTBC will be exposed to multiple stresses including reactive oxygen species, nitric oxide, and acidic conditions when growing in the intracellular environment [32, 33]. The resistance to the harsh intracellular environment is essential for the survival and spread of MTBC. Reactive oxygen intermediates (ROI) and phagosome acidification are the two primary mechanisms for macrophages to degrade intracellular MTB [34]. To determine the effect of Cas1 expression on stress response, we compared the response of two recombination strains to various environmental stresses. The mc^2^ 155_Cas1 strain showed a lower survival rate following exposure to acidic medium (pH 2.5) for 12h when compared with the empty vector control (Figure 3(a)). To determine the effect of Cas1 on oxidative stress response, both recombination strains were treated with H_2_O_2_ for 4h. The mc^2^ 155_Cas1 strain showed a higher sensitivity to oxidative stress induced by H_2_O_2_ treatment (Figure 3(b)). Taken together, these results showed that Cas1 expression increased the sensitivity of* M. smegmatis* to harsh intracellular environment.

### 3.5. Cas1 (Rv2817c) Recombinant* M. smegmatis* Is Hypersensitive to DNA Damaging Agent

**Figure 4:**
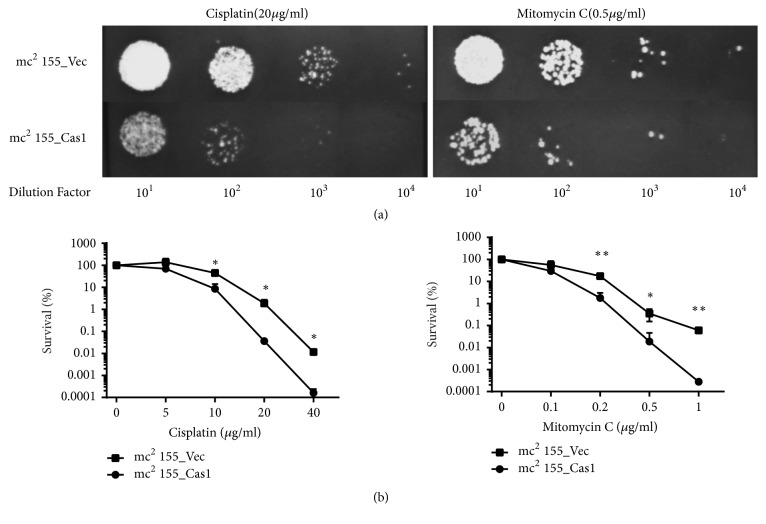
Cas1 impairs the repair of DNA damage. (a) Recombinant strains mc^2^ 155_Cas1 and mc^2^ 155_Vec were serially diluted and plated onto MB 7H10 plates with 20*μ*g/ml Cisplatin or 0.5*μ*g/ml Mitomycin C. Plates were incubated at 37°C for 5 days for observation. (b) Recombinant strains mc^2^ 155_Cas1 and mc^2^ 155_Vec were exposed to various concentration of Cisplatin or Mitomycin C for 2 hours to induce DNA damage and the survival rates were calculated. Error bars indicate standard deviation of triplicate experiments. *∗* P<0.05; *∗∗* P<0.01.

The metal-dependent DNase activity of Cas1 plays a major role in the recombinational repair of DNA double-strand breaks in some genera of bacteria [16, 17]. Although Cas1 in* M. tuberculosis* shares homologous domain architecture with other known endonuclease Cas1 in super-family Cas1_I-II-III, its endonuclease activity has not been observed until now. To determine the potential role of* M. tuberculosis* Type III-A Cas1 in DNA repair, recombinant strains mc^2^ 155_Cas1 and mc^2^ 155_Vec were exposed to DNA damaging agents. Strains mc^2^155_Cas1 showed higher sensitivity to both DNA damaging agents when grown on MB 7H10 plates with Cisplatin (20*μ*g/ml) or Mitomycin C (0.5*μ*g/ml) (Figure 4(a)). Then two recombinant strains were treated with various concentrations of Cisplatin or Mitomycin C for 2 hours to induce DNA damage and then grown on 7H10 agar plates. The surviving fraction of Cas1 recombinant strain was approximately two orders of magnitude lower than empty vector control strain at a high concentration of DNA damaging agent (Cisplatin >5*μ*g/ml, Mitomycin C >0.1*μ*g/ml) (Figure 4(b)). In contrast to the phenomenon of Type I-E Cas1 in* E. coli*, these results indicate that Type III-A Cas1 protein plays a negative role in DNA damage repairing.

### 3.6. Cas1 Recombinant* M. smegmatis* Is More Susceptible to Multiple Antitubercular Agents

**Figure 5:**
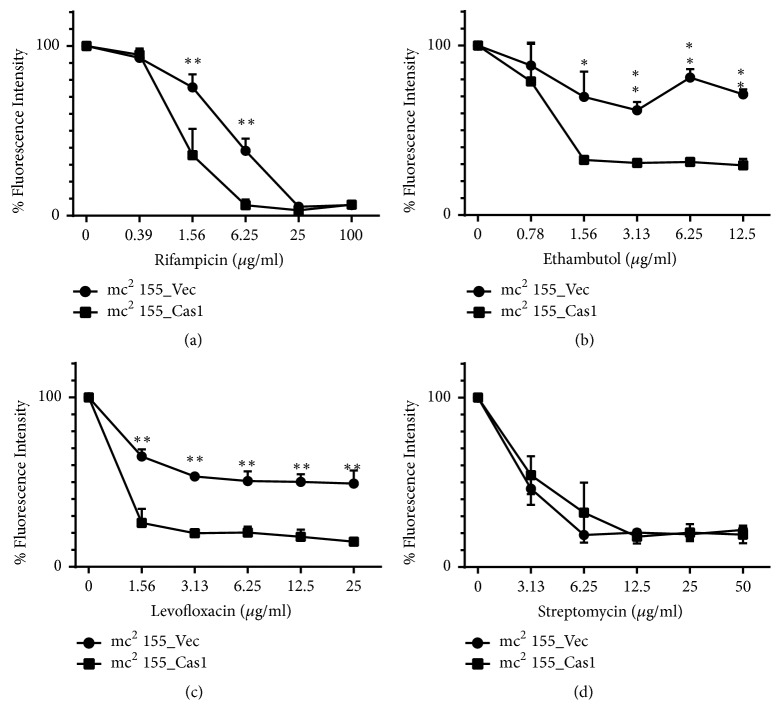
Cas1 increases the sensitivity of M. smegmatis to multiple anti-tuberculosis drugs. REsazurine Microtiter Assay (REMA) was used to determine the antibiotic sensitivity of recombinant strains mc^2^ 155_Cas1 and mc^2^ 155_Vec to different drugs: (a) rifampicin, (b) ethambutol, (c) levofloxacin, and (d) streptomycin. Error bars indicate standard deviation of triplicate experiments. *∗* P<0.05; *∗∗* P<0.01.

Cas1 deletion MTBC strain shows a strong regional bias associated with antibiotic pressure and an increased tendency to be MDR. The REMA assay was used to investigate the effect of Cas1 on mycobacterial drug tolerance. Recombinant strains mc^2^ 155_Cas1 and mc^2^ 155_Vec were exposed to various concentrations of several antimicrobial compounds including isoniazid, rifampin, ethambutol, streptomycin, levofloxacin, and ampicillin as previously described. The sensitivity of mc^2^ 155_Cas1 to rifampin, levofloxacin, and ethambutol was significantly higher than the mc^2^ 155_Vec (Figure 5). However, the sensitivity of mc^2^ 155_Cas1 to streptomycin and the other tested drugs remained similar to mc^2^ 155_Vec (data not shown). All results were confirmed by survival rate assay as previously described. These results indicate that the Cas1 protein contributes to the lethal activity of rifampin, levofloxacin, and ethambutol against wild type cells.

### 3.7. Cas1 (Rv2817c) Expression Reduces Persistence during Drug Treatment

**Figure 6:**
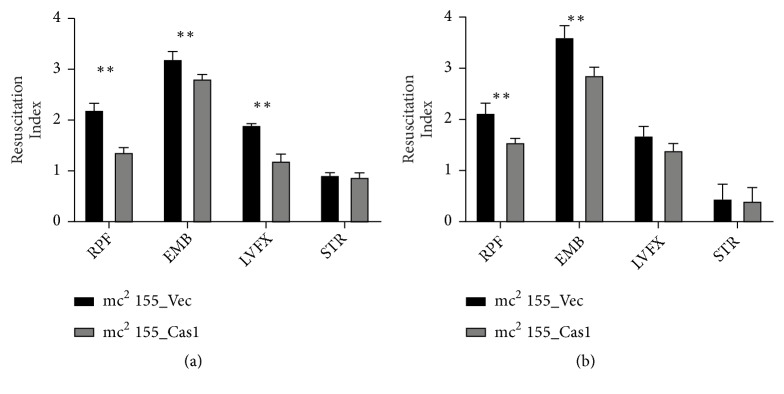
Cas1 expression reduces drug induced persistence. Recombinant strains mc^2^ 155_Cas1 and mc^2^ 155_Vec were exposed to antibiotics (RPF) rifampin 5*μ*g/ml, (EMB) ethambutol 20*μ*g/ml, (LVFX) levofloxacin 20*μ*g/ml, and (STR) streptomycin 20*μ*g/ml for (a) 24h or (b) 48h and then resuscitated by 20nM recombinant RpfE. Resuscitation index was calculated as log10(MPN)-log10(CFU). Error bars indicate standard deviation of triplicate experiments. *∗* P<0.05; *∗∗* P<0.01.

The presence of persister cells that survive in treatment is still one of the major problems during MTBC chemotherapy. MTBC can enter a persistence state in response to an unfavorable external condition or anti-tubercular treatment. The bacilli in the persistence state were in a nonreplicating state and tolerant to the usually lethal effect of antibiotics [35–37]. The persistence of* M. smegmatis* was assessed by using the resuscitation index (RI) calculated as log 10 (MPN) -log 10 (CFU).* M. smegmatis* cultures were treated with different antibiotics for 24h or 48h, respectively. A significant increase of RI after treatment with rifampin, ethambutol, and levofloxacin was observed in the first 24h. Cas1 overexpressing significantly reduced the drug-induced persistence compared with wild-type strain (Figure 6(a)). However, streptomycin hardly resulted in generation of persistence and no statistical difference was observed between two groups. Prolonged incubation of* M. smegmatis* with antibiotics led to an increased RI for ethambutol but not for rifampin, levofloxacin, or streptomycin (Figure 6(b)). In agreement with the result of antimicrobial susceptibility, Cas1 increased* M. smegmatis* sensitivity to antibiotics by reducing the drug-induced persistence.

## 4. Discussion

Although type III-A CRISPR-Cas system in MTBC is predicted to play a similar role with other type CRISPR-Cas systems in phage resistance, the spacer adaptation has not been observed in MTBC so far. We challenged BCG with mycobacteriophage D29 and generated resistant clone to phage. No newly acquired spacer that matches mycobacteriophage D29 genome was found in sequencing reads in this study. Meanwhile, none of the spacer matches other mycobacteriophage genomes can be found in the complete genomes of sequenced MTBC. One of the possible explanations is that a single spacer directed at an intragenic proto-spacer is sufficient to drive phage extinction in type III CRISPR-Cas systems, and having multiple spacers directed at the same invader could instead result in negative fitness [14]. Even though BCG did not acquire new spacers under laboratory conditions, they could still achieve the resistance to mycobacteriophage D29 in a short time. This phenomenon may indicate that the resistance of MTBC against phage may not mainly rely on CRISPR-mediated spacer acquisition.

CRISPR associated protein Cas1 is one of the most conserved DNA endonucleases in CRISPR-Cas systems and often found lost in MTBC. Cas1 mediates the adaptation process in cooperation with another ssRNA-specific endonuclease Cas2 [16, 38]. In addition to anti-viral immunity, Cas1 is also involved in many processes including chromosome segregation and recombinational repair of DNA double-strand breaks [17]. Deletion of Cas1 can lead to the deficiency of acquired immunity against phage and hypersensitivity to DNA damage in some genera of bacteria. The role of Cas1 in MTBC is still not fully understood, but Cas1 is considered to be one of the genes essential for MTBC growth and cholesterol catabolism in vitro [39–41]. Moreover, when exposed to antibiotic or environmental stresses, the transcription of Cas1 gene in MTBC changed accordingly [26], which suggests Cas1 may also be involved in drug resistance and stress response. Interestingly, the Cas1 protein was no longer found in the proteome of an infected guinea pig model in contrast to a vitro model [42, 43]. Also the Cas1 gene is no more indispensable for MTBC H37Rv to survive in an in vivo infection model [44].

MTBC Beijing family, considered to be the only natural instance for CRISPR-containing bacteria without a Cas1 gene, is still a very successful pathogen in high TB load areas and has an increased tendency to be MDR. How likely does MTBC Beijing family benefit from the deletion of Cas1 to make it become a dominant strain in high antibiotic pressure areas? It was noticed that hosts that are frequently infected by phages would disproportionately benefit from having a type III-A system, but the same system might cause loss of fitness in an environment with lower viral load due to the high target promiscuity and resulting cell toxicity [14]. Even though deletion of Cas1 can result in dysfunction of the type III-A CRISPR system, when growing in vivo MTBC will be frequently exposed to multiple antibiotics and harsh environmental stresses but will not face phage challenge any more. When Cas1 was expressed in* M. smegmatis*, the sensitivity of* M. smegmatis* to multiple anti-tuberculosis drugs and harsh environmental stresses increased, which indicates MTBC may achieve a better fitness in vivo by downregulating or losing Cas1.

Studies on other genera of bacteria also demonstrate that mutation or deletion of Cas1 increases the tolerance to antibiotic and lead to the emergence of multidrug-resistant strains [22–25]. MTBC can also achieve a phenotypic resistance to antibiotics by entering a persistence state, which is one of the most crucial reasons for the long course of TB chemotherapy. Previous studies have proved that the persisters of MTBC can be resuscitated by recombinant Rpf which also can be used to assess the amount of persisters [45]. In this study we determined that the Cas1 increases* M. smegmatis *antibiotic sensitivity by reducing the drug-induced persistence by using recombinant RpfE. When treated with rifampin, ethambutol, or levofloxacin, fewer persister cells were observed in Cas1 recombinant* M. smegmatis*. This may further explain how MTBC Beijing family could benefit from Cas1 deletion in high TB load areas. When facing antibiotic or environmental stresses, Cas1 deletion strains are more likely to enter a persistence state or cause a latent infection to ensure survival, which enables the MTBC to further develop the resistant towards antibiotics. The inverse relationship between CRISPR-Cas and antibiotic resistance suggests that the usage of antibiotic may inadvertently select for strains with Cas1 deletion like MTBC Beijing family. Another theory based on the DNA repair function of Cas1 found in* E. coli* indicates that the deletion of Cas1 prevents the gene mutations from being repaired in time and then leads to a gradual accumulation of drug resistance mutations [21]. However, there is still no report of the Cas1 DNA damage repairing function except the Cas1 in* E. coli*. And the expression of Cas1 in* M. smegmatis* did not result in an increased resistance to DNA damage. As an important component of the CRISPR system, the function of Cas1 in some other CRISPR systems has been thoroughly studied, but it is not fully understood yet when it comes to MTBC type III-A CRISPR system. The function of Cas1 is worthy of further study, which can bring a new sight for the treatment of tuberculosis.

## Data Availability

The data used to support the findings of this study are available from the corresponding author upon request.

## Supplementary Materials

Supplementary Materials Suppl. Table 1: primers used in this study. Suppl. Figure 1: detection of Cas1 gene (Rv2817c) in clinical isolates.
