# Supplementary material for: The Mycobacterium tuberculosis CRISPR-Associated Cas1 Involves Persistence and Tolerance to Anti-Tubercular Drugs
Source: Biomed Res Int. 2019 Apr 2;2019:7861695. doi: 10.1155/2019/7861695 (PMC6466960; doi:10.1155/2019/7861695)
Supplement: Supplementary Materials — Suppl. Table 1: primers used in this study. Suppl. Figure 1: detection of Cas1 gene (Rv2817c) in clinical isolates. [file 7861695.f1.zip › mat.7861695.v3.pdf]

Suppl. Table 1. Primers used in this study.

Gene		Sequence
CRISPR loci 1	F	CACCGACACCCCGAACACCAC
	R	CTCAACGCCAGAGACCAGC
CRISPR loci 2	F	TTAGGTCTCGCCTATACCTCCTCGATGAACCACC
	R	TCAGCGCAGAGGAGTTTGTG
Rv2817c-Rv2816c	F	AAAGGTCTCACTTCATGGTGCAGCTGTATGTCTC
	R	TTAGGTCTCGCCTATCAAAAGAACACAAACTCC
EcoCas1	F	TTgaattcATGCATCATCACCATCACCATGTGCAGCTGTATGTCTCG
HindCas1	R	GGaagcttTTAGGCTCCGGATGGCTC

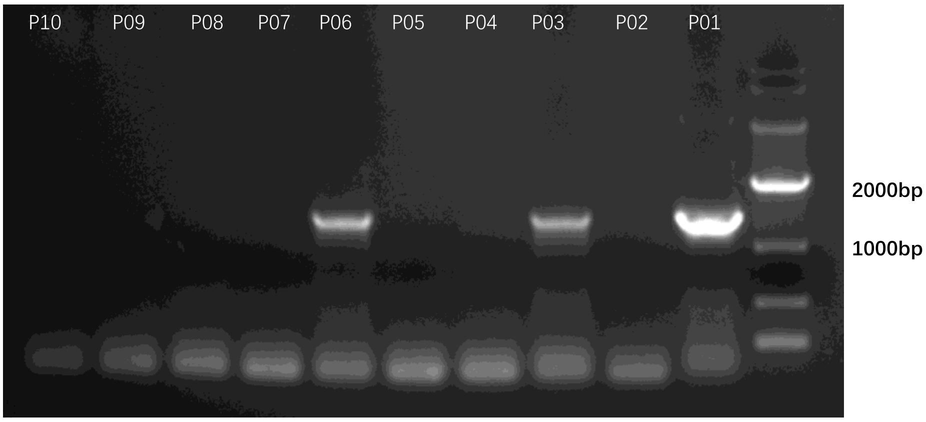

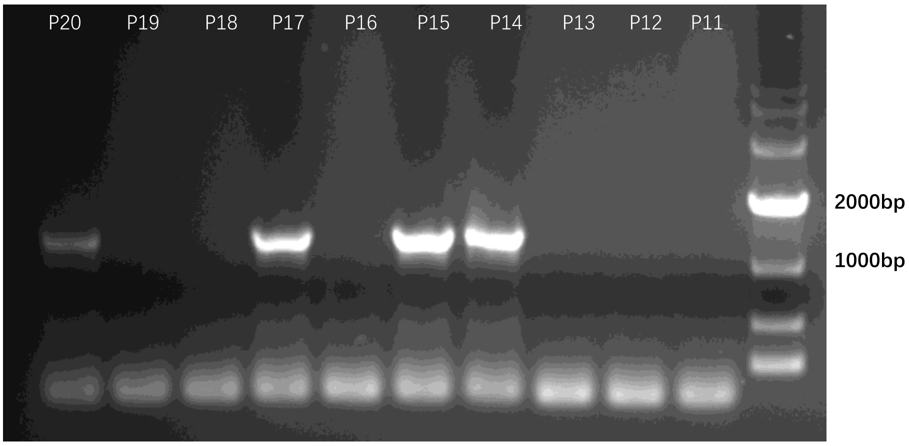

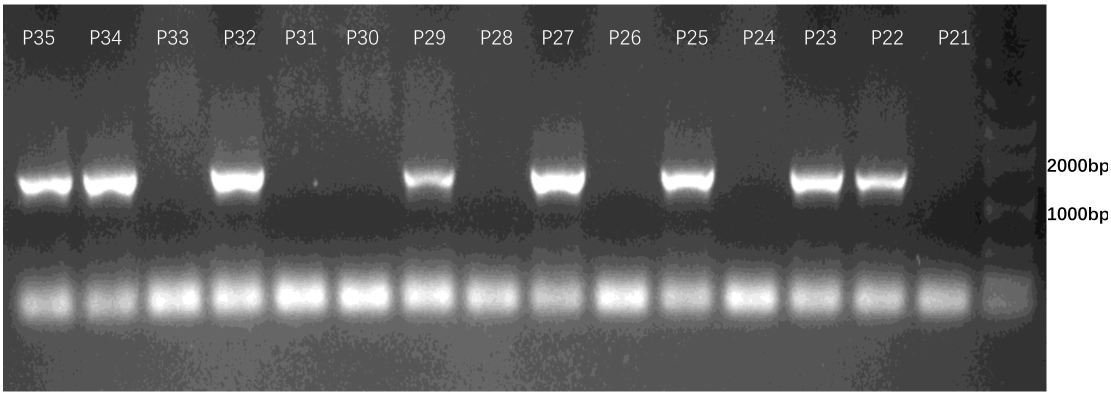

Suppl. Fig. 1. Detection of Cas1 gene (Rv2817c) in clinical isolates
