# Supplementary material for: The Mycobacterium tuberculosis CRISPR-Associated Cas1 Involves Persistence and Tolerance to Anti-Tubercular Drugs
Source: Biomed Res Int. 2019 Apr 2;2019:7861695. doi: 10.1155/2019/7861695 (PMC6466960; doi:10.1155/2019/7861695)
Supplement: Supplementary Materials — Suppl. Table 1: primers used in this study. Suppl. Figure 1: detection of Cas1 gene (Rv2817c) in clinical isolates. [file 7861695.f1.zip › Suppl. Figure 1_BMRI_2710087.docx]

Suppl. Fig. 1. Detection of Cas1 gene (Rv2817c) in clinical isolates
