# Supplementary material for: The Mycobacterium tuberculosis CRISPR-Associated Cas1 Involves Persistence and Tolerance to Anti-Tubercular Drugs
Source: Biomed Res Int. 2019 Apr 2;2019:7861695. doi: 10.1155/2019/7861695 (PMC6466960; doi:10.1155/2019/7861695)
Supplement: Supplementary Materials — Suppl. Table 1: primers used in this study. Suppl. Figure 1: detection of Cas1 gene (Rv2817c) in clinical isolates. [file 7861695.f1.zip › Suppl. Table 1_BMRI_2710086.docx]

Suppl. Table 1. Primers used in this study.

| Gene |  | Sequence |
| --- | --- | --- |
| CRISPR loci 1 | F | CACCGACACCCCGAACACCAC |
|  | R | CTCAACGCCAGAGACCAGC |
| CRISPR loci 2 | F | TTAGGTCTCGCCTATACCTCCTCGATGAACCACC |
|  | R | TCAGCGCAGAGGAGTTTGTG |
| Rv2817c-Rv2816c | F | AAAGGTCTCACTTCATGGTGCAGCTGTATGTCTC |
|  | R | TTAGGTCTCGCCTATCAAAAGAACACAAACTCC |
| EcoCas1 | F | TTgaattcATGCATCATCACCATCACCATGTGCAGCTGTATGTCTCG |
| HindCas1 | R | GGaagcttTTAGGCTCCGGATGGCTC |
